# Computational investigation of a new pyridin-2-yl isomer of thiophene–pyridine 1,3,4-oxadiazole (MTPO) for steel corrosion inhibitor design

**DOI:** 10.1007/s11356-026-38362-y

**Published:** 2026-10-30

**Authors:** Heshani Balasooriya, Chunqing Li, Feng Wang

**Affiliations:** 1 School of Science, Computing and Emerging Technologies, Swinburne University of Technology, Melbourne, Victoria 3122 Australia; 2 School of Engineering, RMIT University, Melbourne, Victoria 3000 Australia

**Keywords:** Iron corrosion inhibition, 1,3,4-Oxadiazole corrosion inhibitors, Density functional theory (DFT), Electronic structure descriptors, Corrosion inhibitor design

## Abstract

This study develops a computational framework for the rational design of corrosion inhibitors for iron surfaces, using the reported inhibitor 2-(5-methylthiophen-2-yl)-5-(pyridin-3-yl)-1,3,4-oxadiazole (MTPO-3) as a reference. Systematic density functional theory (DFT)-based (B3LYP/6-311 ++ G(d,p)) calculations identify its positional isomer, 2-(5-methylthiophen-2-yl)-5-(pyridin-2-yl)-1,3,4-oxadiazole (MTPO-2), as the lowest energy isomer, more stable than MTPO-3 by 0.57 kJ · mol^−1^. The computational method is validated by reproducing experimental ^1^H NMR chemical shifts of MTPO-3 with a small RMSD of 0.30 ppm. Comparative ^13^C NMR analysis reveals pronounced chemical shift changes localised on the pyridine ring (P-ring), indicating positional isomer induced electronic redistribution. Time-dependent DFT calculations show that MTPO-2 exhibits a red shifted absorption maximum (λ_max_ = 345.0 nm) relative to MTPO-3 (λ_max_ = 341.71 nm), enhanced oscillator strength, and an additional absorption band at ~ 281 nm absent in MTPO-3. Frontier orbital analysis indicates that while the HOMOs of both isomers are dominated by the thiophene and oxadiazole units, the LUMOs possess substantial P-ring character, imparting partial charge transfer (CT) character to the lowest energy excitations. Ionisation potential (IP) spectra computed using the outer valence Green’s function (OVGF) method further reveal systematically lower binding energies for MTPO-2, with strong spectral correlation to MTPO-3 (RMSD = 0.175 eV), demonstrating that nitrogen repositioning reshapes the occupied electronic manifold through conjugated aromatic coupling. These results show that P-ring positional isomerism modulates both occupied and unoccupied electronic states with clear spectroscopic signatures, providing electronic structure descriptors relevant to inhibitor design. Detailed adsorption studies on iron surfaces are beyond the scope of this work and will be reported separately.

**Graphical abstract:**

**Figure:**
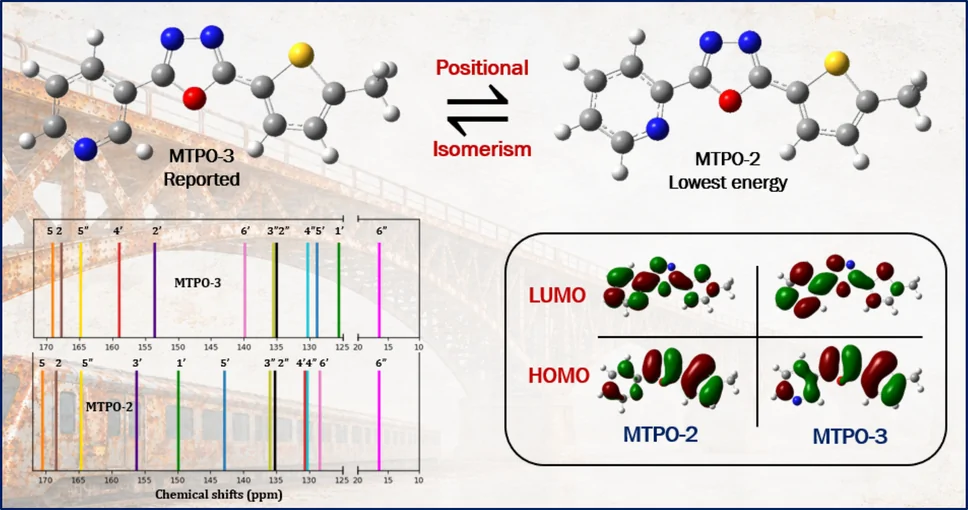

**Supplementary information:**

The online version contains supplementary material available at https://doi.org/10.1007/s11356-026-38362-y.

## Introduction

Corrosion is a common industrial challenge that leads to the gradual deterioration of materials through chemical or electrochemical reactions (Chen et al. 2020; Yadav et al. 2025). Iron corrosion, specifically the rusting of steel, occurs when iron reacts with oxygen and moisture (Chen et al. 2020; Arachchige et al. 2025) to form destructive oxide and hydroxide compounds (Arachchige et al. 2025). This process compromises the mechanical properties and structural integrity of infrastructure, leading to significant economic and environmental consequences (Verma et al. 2021; Balasooriya et al. 2025). While various protection strategies exist, ranging from barrier coatings (Chhipa et al. 2024; Hedayatzadeh et al. 2025) and cathodic protection (Hu et al. 2024; Martinelli-Orlando et al. 2024) to advanced material treatments (Liu et al. 2025; Yadav et al. 2025) organic corrosion inhibitors are widely recognised as one of the most effective and economically viable solutions for protecting steel in aggressive environments.

Among organic inhibitors, heterocyclic compounds containing nitrogen, oxygen, sulphur, and aromatic rings have attracted considerable attention (El Arrouji et al. 2020; Adlani et al. 2024; Guo et al. 2024; Onyeachu et al. 2024; Singh et al. 2024; Timoudan et al. 2024; Belal et al. 2025; Üstün et al. 2025). These molecules effectively mitigate corrosion by adsorbing onto the metal surface, forming a protective barrier that blocks active corrosion sites. Oxadiazoles, a class of five-membered heterocycles containing two nitrogen atoms and one oxygen atom, are particularly effective (Table S1) (Sharma et al. 2024). Their electron-rich heteroatoms and conjugated *π* system facilitate strong interaction with vacant Fe d orbitals via *σ* donation and *π* back-donation mechanisms (Verma et al. 2025). Furthermore, oxadiazoles possess the ability to displace adsorbed water molecules from the metal surface, thereby minimising localised corrosion (Sharma et al. 2023).

**Fig. 1:**
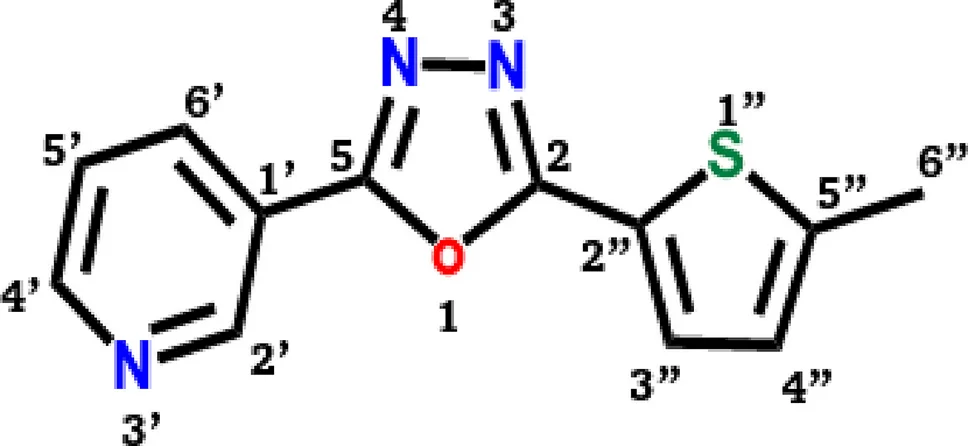
Chemical structure and the nomenclature of a recently synthesised thiophene-pyridine based 1,3,4-oxadiazole hybrid (MTPO-3, here 3 indicates the position of N in P-ring) corrosion inhibitor (Sharma et al. 2025)

Recently, Sharma et al*.* reported 2-(5-methylthiophen-2-yl)−5-(pyridin-3-yl)−1,3,4-oxadiazole (abbreviated as MTPO in the original study and denoted as MTPO-3 in the present work; Fig. 1) as a high performance inhibitor, demonstrating an exceptional inhibition efficiency of 99.05% at 500 ppm in 1 N HCl (Sharma et al. 2025). This superior performance is attributed to its multivalent electron donation framework, where the electron donating thiophene group enhances *π* electron availability and strengthens bonding to the steel surface (Ekerenam et al. 2025). However, the structural flexibility of MTPO leads to multiple isomeric states, including rotational conformers due to single bond connectivity and distinct positional isomers arising from nitrogen position variations within the pyridine ring (P-ring). Although these structural variations produce only modest differences in molecular stability, they can significantly modify the electronic distribution and molecular reactivity (Arulaabaranam et al. 2021; Verma et al. 2024). Such changes may influence interactions with iron surfaces. Therefore, identifying the most stable isomeric forms and understanding how positional isomerism modifies the electronic structure of MTPO is important for corrosion inhibitor design.

To address this challenge, in silico studies based on density functional theory (DFT) offer a powerful approach to explore molecular attributes at the atomic level (Li et al. 2024; Balasooriya et al. 2025). Computational modelling enables detailed characterisation of reactive sites and electronic properties that are often difficult to isolate experimentally (Liu et al. 2022; Ekeocha et al. 2025). While direct experimental validation of all simulation parameters can be challenging, the consistently strong agreement between DFT predictions and experimental benchmarks provides high confidence in these molecular insights.

In this work, we first validate our computational approach by reproducing the experimental ^1^H nuclear magnetic resonance (NMR) and UV–Vis spectra of the reported (MTPO-3) positional isomer (Sharma et al. 2025), confirming the consistency between the experimental structure and its DFT optimised isomer. We then systematically explore the positional isomers and associated rotational conformers of MTPO-3 and identify MTPO-2 as the lowest energy form. Building on this foundation, a detailed comparative analysis of MTPO-3 and MTPO-2 is conducted, including relative energies, NMR and UV–Vis spectroscopic signatures, ionisation potentials, and other electronic properties such as dipole moment and HOMO–LUMO energy gap. The objective of this work is to establish how nitrogen position variation modifies the molecular electronic structure and spectroscopic behaviour of MTPO, thereby providing molecular-level insight into structure–property relationships relevant to corrosion inhibitor design.

## Computational details

In the present work, we systematically examine possible MTPO (Fig. 1) isomers (positional isomers) in isolation. All generated isomers were fully optimised in the gas phase at the B3LYP/6–311 ++ G(d,p) level, which has been widely adopted for corrosion inhibitor studies (Mustafa Mamad et al. 2024; Chraka et al. 2025). Frequency calculations confirmed that all optimised structures correspond to true minima on the potential energy surface, with no imaginary frequencies observed. The original experimentally reported MTPO and the lowest energy isomer are labelled as MTPO-3 (Sharma et al. 2025) and MTPO-2 for their N-positions in the P-ring, 3 or 2, respectively, in later comparative study.

The relative stability of the optimised structures was ranked by their Gibbs free energies. Then, two independent potential energy scans (PES) were performed by rotating the C1′–C5 and C2–C2″ single bonds (see Fig. 1) using the same DFT model. MTPO-2 and MTPO-3 (Sharma et al. 2025) were subsequently reoptimised in chloroform (CHCl_3_) solvent using the solvation model based on density (SMD) (Marenich et al. 2009), an implicit solvation model, which treats the solvent as a homogeneous polarisable medium and is commonly applied to evaluate solvent effects on structural and electronic properties (Gabrielyan et al. 2026). No imaginary frequencies were detected, indicating that all isomer structures represent stable true minimum structures on their potential energy surfaces. Based on the optimised geometries, spectroscopic properties including ^1^H and ^13^C NMR chemical shifts and ultraviolet–visible (UV–Vis) absorption spectra were then calculated for both isomers. NMR chemical shielding (σ) which were computed with the gauge invariant atomic orbital (GIAO) method (Ebrahimi et al. 2011; Xin et al. 2017) are employed to calculate the chemical shifts (δ) of ^1^H and ^13^C NMR spectra, δ = σ(TMS)–σ (MTPO), where σ(TMS) references to tetramethylsilane (TMS) at 182.47 ppm for ^13^C and 31.88 ppm for ^1^H. The UV–Vis absorption spectra are calculated using TD-DFT method up to 60 excited state transitions using the same B3LYP/6–311 ++ G(d, p) level of theory. Finally, the ionisation potentials of MTPO-2 and MTPO-3 are calculated using outer valence Green’s function (OVGF) with the 6–311 ++ G basis set. The pole strength (PS) of the transitions collected is larger than 0.85 to ensure the validation of the single electron approximation.

All calculations are implemented in the Gaussian 16 (Frisch et al*.* 2016) package on Swinburne’s OzSTAR supercomputing cluster.

## Results and discussion

### The MTPO isomers

**Fig. 2:**
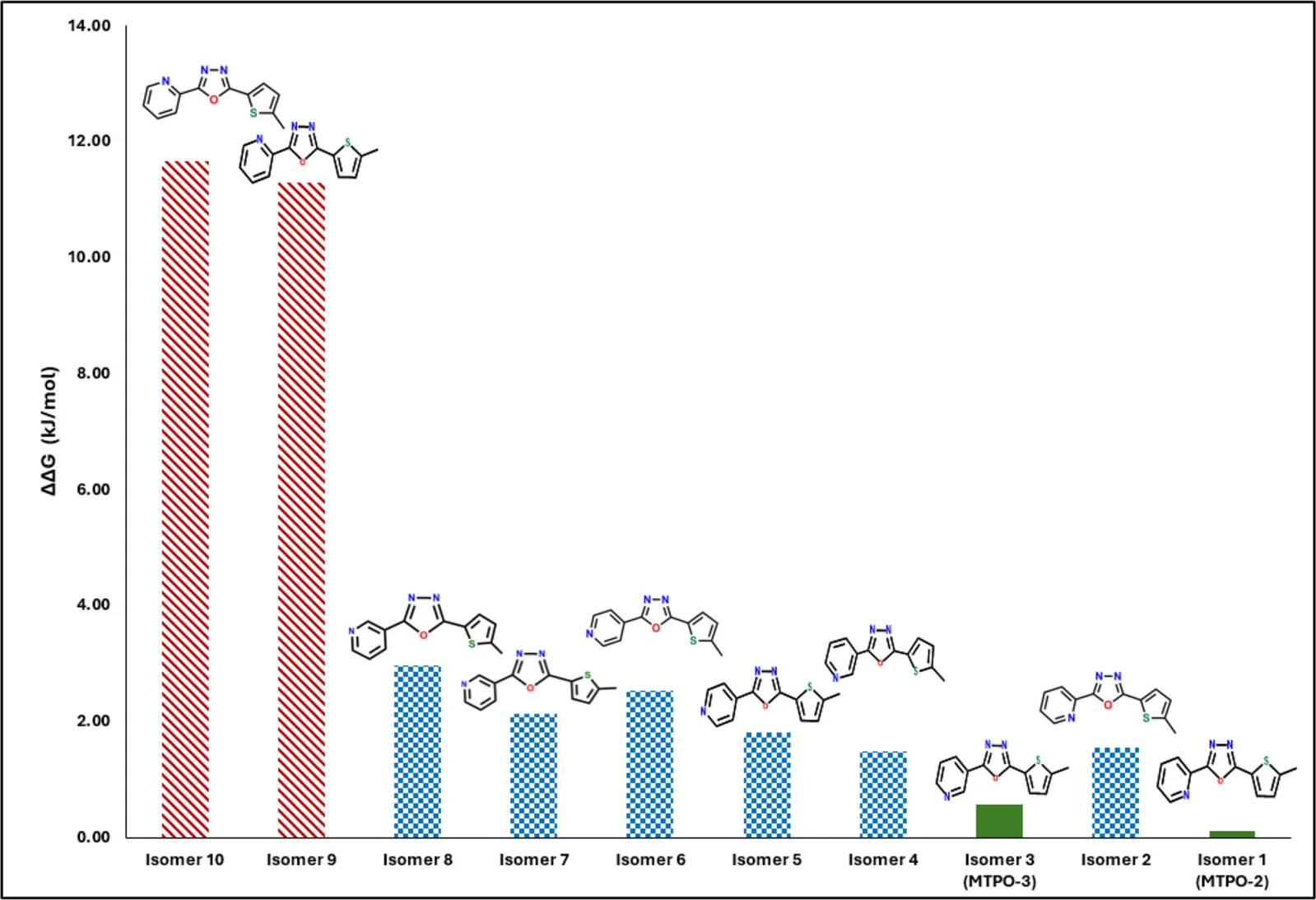
Stability of the obtained isomers of MTPO with respect to their ∆∆G (kJ · mol⁻^1^) using B3LYP/6–311 ++ G(d,p) method. Here, MTPO-3 (Isomer3) is the reported MTPO inhibitor by Sharma et al. (2025), whereas MTPO-2 (Isomer1) is the lowest energy isomer

A total of ten stable isomers of the MTPO inhibitor (Sharma et al. 2025) sharing the 1,3,4-oxadiazole core are obtained using DFT calculations. Figure 2 compares the stability (relative Gibbs free energies (ΔΔG)) of the 10 MTPO isomers, Isomer1, Isomer2, …, Isomer10, revealing a clear stability hierarchy that is governed by the orientation, interaction, and delocalisation of heteroatom lone pairs within the conjugated framework. The 10 stable isomers shown in Fig. 2 form five pairs according to the N-position in the P-ring (2′–6′), representing five positional isomers. Within each pair, the two structures are rotational conformers that differ in the orientation of the thiophene ring. The isomers are grouped into three clusters: the low energy isomers (Isomer1 and Isomer3, green); mid energy isomers (Isomer2, 4–8, blue) and high energy isomers (Isomer9 and Isomer10, red). The energy landscape spans a wide range, from low lying structures within ~1 kJ mol⁻^1^ (green) to highly destabilised isomers exceeding 10 kJ mol⁻^1^ (red). Low energy Isomer1 and Isomer3 are characterised by favourable lone pair alignment that preserves molecular planarity and conjugation across the pyridine–oxadiazole–thiophene scaffold. That is, the N atom locates on the 2′- and 3′-positions of the P-ring. In contrast, higher energy structures such as Isomer9 and Isomer10 arise when the lone electron pairs of Ns are oriented close to each other or toward congested regions of the molecule, leading to increased Pauli repulsion and disruption of π electron delocalisation. These electronic penalties manifest as significant increases in ΔΔG, highlighting the sensitivity of Isomer1 (MTPO-2) and Isomer3 (MTPO-3) stability to minor changes in heteroatom positioning and ring orientation. In these two low energy Isomers, the P-ring N atoms at the 2′- and 3′-positions are both far away from the possible lone electron pairs of N and O on the O-ring, while the thiophene ring oriented in the same direction. Between these two low energy forms, the greater stability of MTPO-2 relative to MTPO-3 can be attributed to the position of the P-ring nitrogen at the 2′-site, which lies closer to the oxadiazole linking carbon and thereby permits more effective π conjugation and orbital overlap between the P-ring and the oxadiazole core, consistent with the enhanced electronic coupling identified from the frontier orbital and UV–Vis analyses in Section “UV–Vis Spectra of the MTPO Isomers”. In MTPO-3, the more distal 3′-position of the nitrogen atom reduces this conjugative interaction.

A distinct group of high energy isomers, Isomer9 and Isomer10, is observed, which lie more than ~ 10 kJ mol⁻^1^ above the global minimum. The isomers with the N-position at 6′-P-ring that the lone electron pair of the N on the P-ring disrupt planarity or introduce steric congestion near the oxadiazole core are strongly destabilised. These large ΔΔG values indicate severe energetic penalties, rendering these structures thermodynamically inaccessible under normal conditions. Such destabilisation is consistent with unfavourable steric interactions and reduced electronic delocalisation, and these isomers are therefore excluded from further consideration.

Several isomers fall into an intermediate stability range (approximately 2–4 kJ mol⁻^1^ above the minimum), including Isomer2 and Isomers4–8. These structures are moderately destabilised relative to the lowest energy forms but may still be populated at finite temperature. However, their higher ΔΔG values suggest that they are less likely to dominate inhibitor behaviour and are secondary to the lowest energy configurations. Based on this stability analysis, MTPO-2 and MTPO-3 are selected for subsequent detailed studies. In particular, potential energy surface scans (PES) will be performed to further elucidate their conformational flexibility and rotational energy barriers, providing deeper insight into the structural factors that may influence their electronic properties and subsequent adsorption behaviour.

**Fig. 3:**
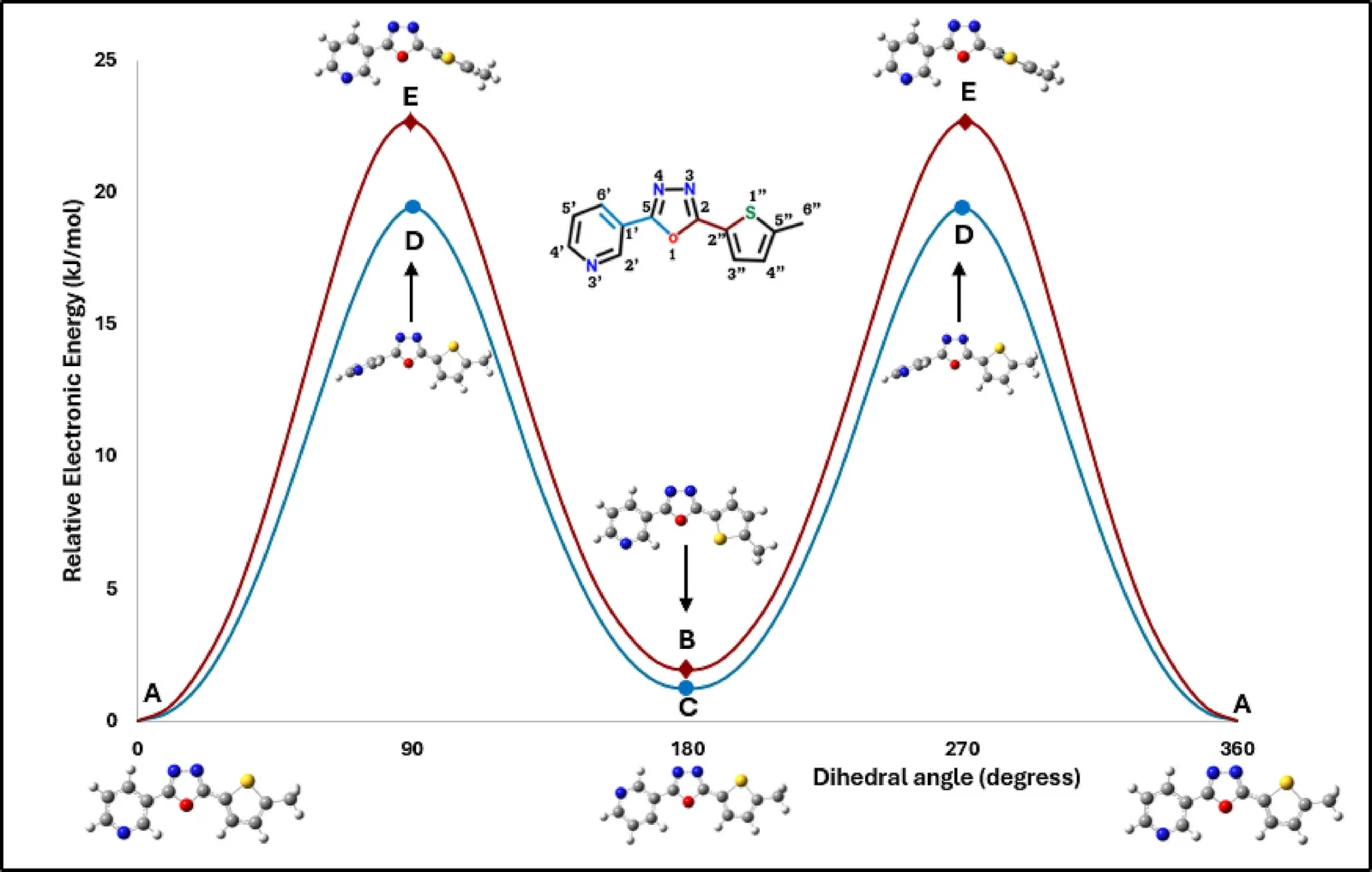
Potential energy scans (PES) of the optimised MTPO-3 (the reported (Sharma et al. 2025)) molecule at the B3LYP/6–311 ++ G(d,p) level of theory in gas phase. The blue curve and red curve are the PES of rotating the respective bond marked in the structure

As shown in Fig. 2, the reported MTPO-3 (2-(5-methylthiophen-2-yl)−5-(pyridine-3-yl)−1,3,4-oxadiazole, Isomer3) (Sharma et al. 2025) is in fact not the lowest energy isomer. It is 0.57 kJ · mol⁻^1^ (ΔΔG) less stable than the lowest energy MTPO-2 (2-(5-methylthiophen-2-yl)−5-(pyridine-2-yl)−1,3,4-oxadiazole, or Isomer1) where the N locates on position 2 of P-ring. We further performed the PES of MTPO-3 via rotating around the rotatable single bonds C1′-C5 and C2-C2″), respectively, over 360° (Fig. 3). The resulting energy profiles reveal well-defined minima and barriers that reflect the interplay between conjugation, steric effects, and heteroatom lone-pair interactions in this near-planar heteroaromatic system. For both rotational coordinates, the PES exhibits a pair of nearly symmetric profiles, with equivalent energy barriers located at dihedral angles close to 90° and 270°, and a reflection plane at 180°. This symmetry is consistent with the underlying molecular geometry and the repetitive electronic environment encountered during full bond rotation. In both scans, the global minimum (conformation A) occurs near 0° (and equivalently 360°), corresponding to a near-planar arrangement in which conjugation across the pyridine–oxadiazole–thiophene framework is maximised.

Rotation about the C1′–C5 bond (blue curve) connects conformations A–D–C–D–A. Starting from the most stable conformation A (MTPO-3), the energy rises steadily to a maximum at D, reaching a barrier of approximately 19.3 kJ mol⁻^1^ near 90°, before decreasing to a shallow local minimum C at 180°, lying only 1.22 kJ mol⁻^1^ above the global minimum (A, i.e. MTPO-3). Conformation C represents an alternative low energy arrangement in which the oxadiazole oxygen and thiophene sulfur are located on the same side of the molecule. As shown in Fig. 3, at 90° (and 270°), one of the terminal rings, P-ring or T-ring, is approximately perpendicular to the middle O-ring.

Similarly, the red curve is produced through rotating the C2–C2″ (see the bonds coloured in red), connecting A–E–B–E–A in Fig. 3. The energy barriers at point E reach 22.67 kJ · mol⁻^1^ at dihedral angles at 90° and 270°, respectively, implying more restricted rotational flexibility and significant torsional strain in these regions. The symmetry of the energy profile reflects the planarity of reported MTPO-3. The high energy barriers observed along the C2–C2′ rotation primarily originate from the disruption of π conjugation between the P-ring and O-ring, which destabilises the delocalised electronic structure of MTPO-3. This effect is reinforced by unfavourable lone pair–lone pair Pauli repulsion involving the pyridine nitrogen and oxadiazole heteroatoms at twisted geometries. As a result, the PES scans of MTPO-3 indicate that the inhibitor strongly favours a near-planar geometry, which is important for maintaining conjugation and stabilising electronic structure, whereas transitions to B or C require overcoming moderate energy barriers of approximately 20 kJ · mol⁻^1^, limiting conformational interconversion under ambient conditions.

The major structural difference between the MTPO-3 and MTPO-2 isomer pair is the position of the nitrogen atom on 3′ (MTPO-3) or 2′ (MTPO-2) positions of the P-ring, so that MTPO-2 is slightly (0.57 kJ · mol^−1^) more stable than the reported MTPO-3 (Sharma et al. 2025). This study seeks to clarify how structural variations among MTPO isomers translate into property differences relevant to iron surface inhibition, with the goal of informing future rational inhibitor design.

### Positional isomer induced NMR chemical shifts

The ^1^H NMR chemical shifts of MTPO-3 were calculated in CDCl_3_ and compared with the experimental measurements reported by Sharma *et al.* in the same solvent (Table 1) (Sharma et al. 2025). The resulting root-mean-square deviation (RMSD) of 0.30 ppm indicates that the calculations reproduce the experimental data with good accuracy. This level of agreement is consistent with previous studies demonstrating that the B3LYP functional provides reliable ^1^H NMR predictions for heterocyclic compounds (Guzzo et al. 2018; Nguyen 2021). Accordingly, the same DFT method was employed to calculate the ^1^H NMR chemical shifts of MTPO-2, as summarised in Table 1. The O-ring of MTPO-3 (Fig. 1) contains no hydrogen atoms. Of the eight hydrogen atoms in MTPO, four are located on the P-ring (H′) and four on the thiophene ring (H″). The isomerism between MTPO-3 and MTPO-2 arises solely from the nitrogen position within the P-ring. Consequently, small upfield shifts are observed for the P-ring protons upon isomerisation, whereas the T-ring protons remain largely unaffected, with the exception of H3″, which exhibits a minor shift of approximately 0.05 ppm.

**Table 1:** Comparison of the calculated ^1^H NMR chemical shifts (ppm) and the available experimental data in CDCl_3_ solvent for MTPO-3 and MTPO-2*

Atom site	MTPO-3		MTPO-2	Δ(2–3)
	B3LYP	Exp^#^	B3LYP	
H 2′	9.56	9.30	9.02	−0.54
H 6′	9.01	8.77	8.79	−0.22
H 4′	8.99	8.44–8.32	8.18	−0.81
H 3″	7.92	7.64	7.97	0.05
H 5′	7.74	7.47	7.68	−0.06
H 4″	7.07	6.85	7.07	0.0
H 6″*2	2.73	2.56	2.73	0.0
H 6″	2.67	2.56	2.67	0.0
**RMSD**	**0.30**	**-**	**0.35**^**&**^	

*Both with 6–311 ++ G(d,p) basis set in CHCl_3_ solvent

^#^The experimental results for MTPO-3 (Sharma et al. 2025)

^&^RMSD between MTPO-3 and MTPO-2 based on B3LYP calculations

**Fig. 4:**
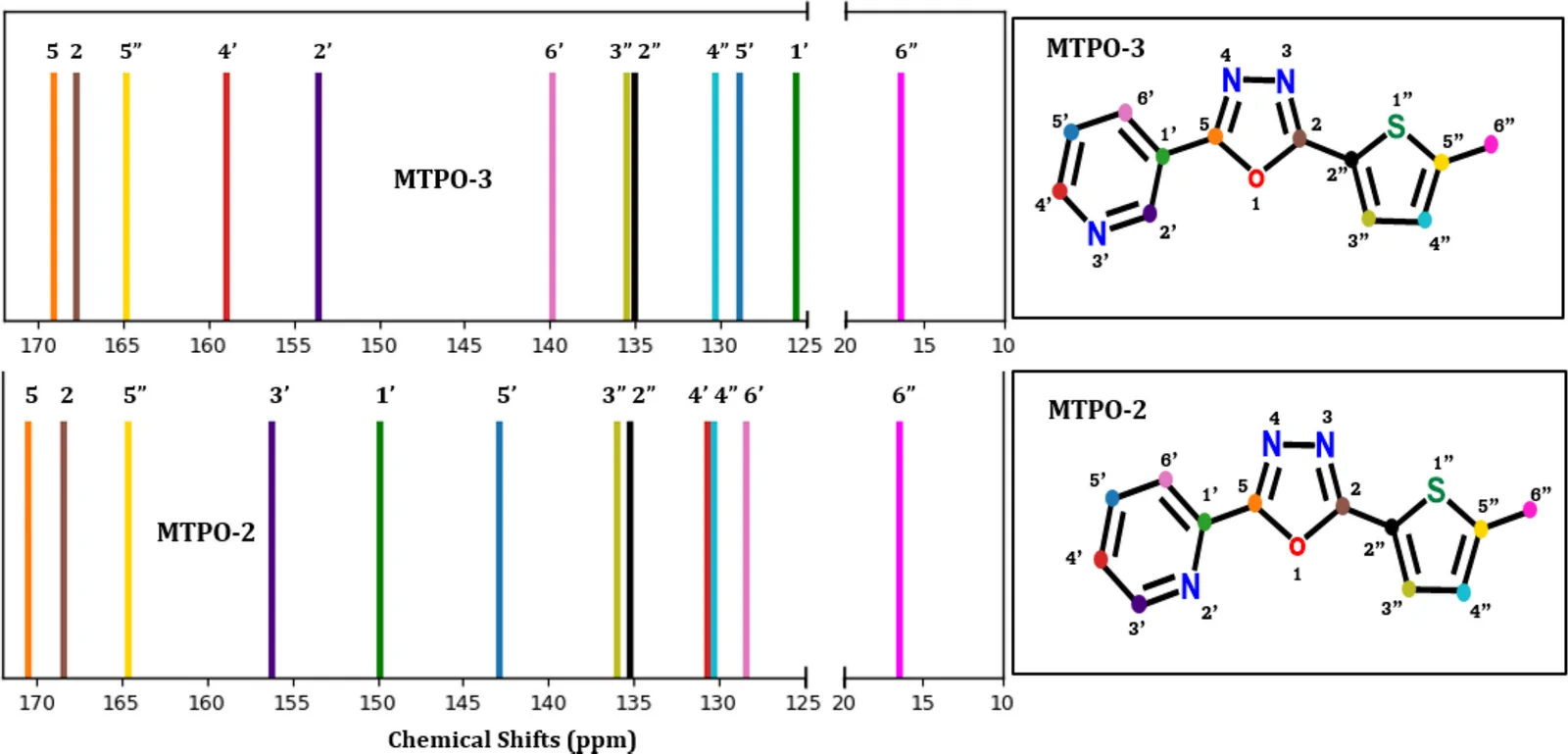
Comparison of the calculated ^13^C NMR chemical shifts for MTPO-2 and MTPO-3, using the B3LYP/6–311 ++ G(d,p) level of theory in CHCl_3_ solvent

Figure 4 compares the ^13^C NMR chemical shifts of MTPO-2 and MTPO-3, which span a wide spectral range from approximately 15 to 180 ppm. With the exception of the methyl carbon resonance observed near 16 ppm, all remaining carbon signals lie within the aromatic region (130–180 ppm), consistent with the highly conjugated heteroaromatic framework of both compounds. The ^13^C NMR spectra clearly demonstrate that the MTPO-2 and MTPO-3 are distinct chemical species, despite possessing the same molecular formula and number of carbon atoms.

Although both positional isomers contain the same number of carbon centres, there is no strict one-to-one correspondence between individual carbon atoms in MTPO-2 and MTPO-3. This arises because positional isomerisation involves relocation of the nitrogen atom within the P-ring, which fundamentally alters the electronic environment and bonding context of the surrounding carbons. Consequently, a given carbon in MTPO-3 does not map directly onto an equivalent carbon in MTPO-2; for example, C2′ in MTPO-3 is not electronically equivalent to C3′ in MTPO-2, even though both reside on the pyridine ring.

Several general trends can be identified from the spectra. First, carbons directly bonded to more electronegative atoms, such as nitrogen, oxygen, or sulfur, exhibit downfield shifts (deshielding) due to reduced local electron density. For example, C2, C5 in O-ring; C5″ in T-ring, C2′/C3′ and C4′/C1′ in P-ring of the positional isomers downfield in the region of δ_C_ ≥ 150 ppm. This effect is most pronounced for carbons adjacent to the pyridine nitrogen, whose electron-withdrawing character strongly perturbs the π electron distribution of the ring. As the nitrogen atom moves from the 3′-position in MTPO-3 to the 2′-position in MTPO-2, the pattern of deshielding and shielding across the pyridine carbons is reorganised accordingly. Second, the impact of positional isomerism is highly localised to the P-ring. For example, large chemical shifts among the carbons in the positional isomers are C4′, C1′, C5′, and C6′, all in the P-ring for over ± 10 ppm shifts. The largest δ_C_ shift is C4′ for − 28.33 ppm, followed by C1′ for 24.32 ppm and C5′ for 13.98 ppm and C6′ for − 11.51 ppm. The pyridine carbons display substantial shift changes, reflecting the sensitivity of this ring to the position of the nitrogen atom and its associated lone pair. The carbon resonances associated with the T-ring and the O-ring show only minor chemical shift variations between MTPO-2 and MTPO-3, indicating that their electronic environments are largely preserved upon nitrogen repositioning.

The positional isomer dependent changes observed in the ^13^C NMR spectra directly reflect redistribution of π electron density within the conjugated framework, particularly across the P-ring where nitrogen repositioning induces pronounced shielding and deshielding effects. These local variations in carbon chemical shifts indicate altered participation of pyridine carbons in the frontier molecular orbitals, consistent with changes in HOMO and LUMO localisation and energies. Accordingly, the ^13^C NMR results provide a spectroscopic manifestation of the electronic reorganisation that underpins the frontier orbital analysis discussed below.

### UV–Vis spectra of the MTPO isomers

**Fig. 5:**
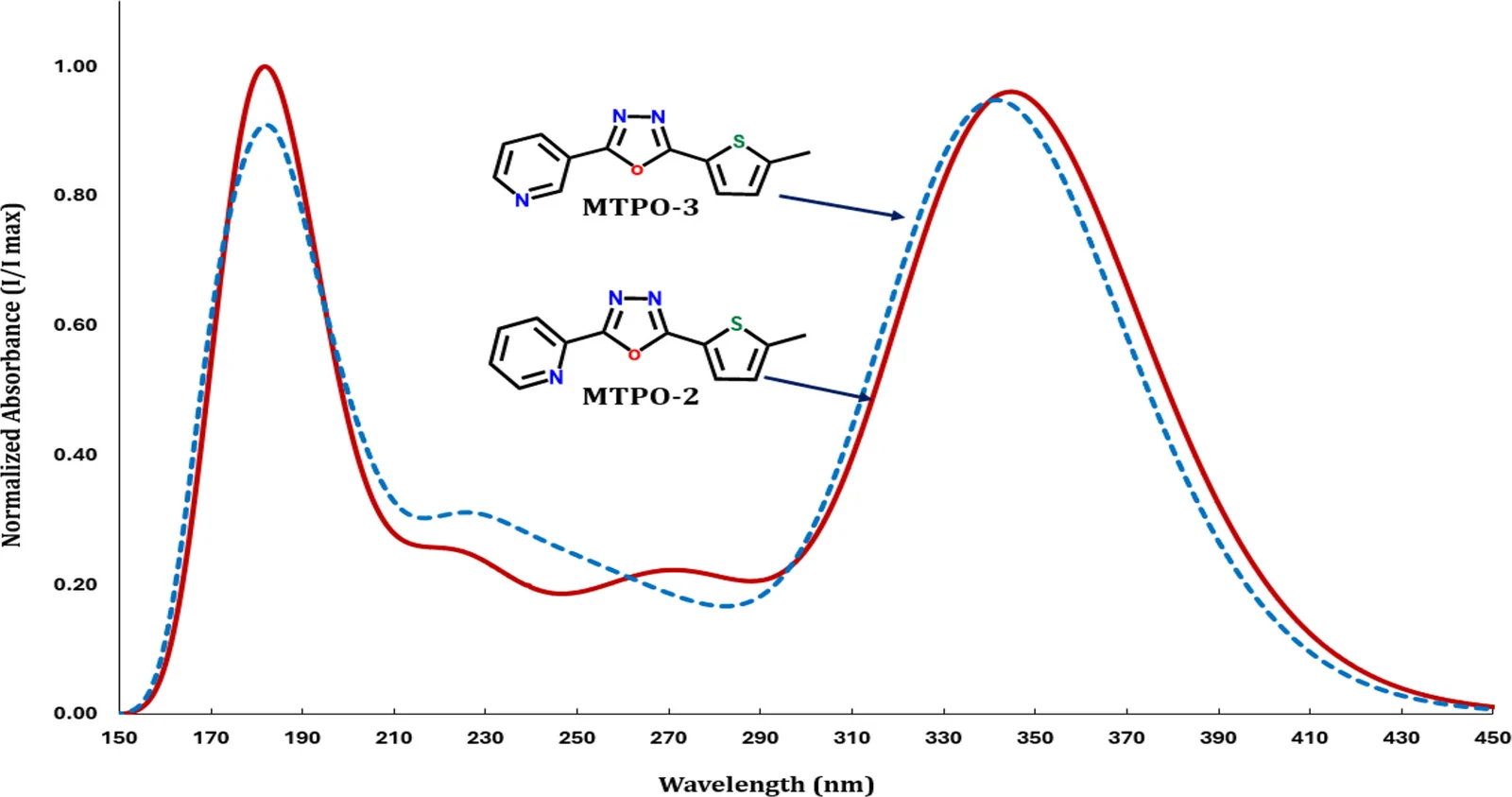
Comparison of the UV–Vis spectra for MTPO-3 and MTPO-2 isomers, calculated using B3LYP/6–311 ++ G (d,p) level of theory in CHCl_3_ solvent

The simulated UV–Vis spectra of MTPO-2 and MTPO-3 exhibit clear and systematic differences in both absorption intensity and overall spectral profile, despite both positional isomers absorbing predominantly in the ultraviolet region. Figure 5 presents the UV–Vis spectra calculated using TD-DFT at the B3LYP/6–311 ++ G(d,p) level in CHCl_3_. Across the examined wavelength range, MTPO-2 consistently displays higher absorbance than MTPO-3. This enhanced intensity reflects a larger oscillator strength for the dominant electronic transitions rather than the presence of an extended chromophoric system. Instead, the difference arises from more effective electronic coupling between the P-ring and the oxadiazole core in MTPO-2, where nitrogen repositioning at the 2′ position promotes increased delocalisation of the frontier orbitals and enhanced highest occupied molecular orbital (HOMO) and lowest unoccupied molecular orbital (LUMO), i.e. HOMO–LUMO energy overlap. Consequently, the stronger absorption of MTPO-2 originates from increased transition probability driven by P-ring electronic participation, rather than from global extension of the conjugated framework.

Although MTPO-2 and MTPO-3 share the same thiophene–oxadiazole backbone, their UV–Vis spectra reveal subtle but reproducible differences that originate from the distinct electronic role of the P-ring in the two positional isomers. Both compounds exhibit a principal absorption band in the near UV region; however, MTPO-2 shows a slight bathochromic shift, with its absorption maximum at λ_max_ = 345.0 nm compared to λ_max_ = 341.71 nm for MTPO-3. Similar subtle wavelength shifts have been reported for closely related molecular conformers, where interpretation relied on the complete spectral signature rather than λmax alone (Khattab et al. 2016; Wang 2023). In addition to this small shift, differences in band shape and intensity are observed in the higher energy regions spanning approximately 160–200 nm, 210–240 nm, and 260–300 nm. These more pronounced variations can be directly attributed to nitrogen repositioning within the P-ring, which modifies the electronic coupling between the pyridine ring and the oxadiazole core. In MTPO-2, placement of the nitrogen atom at the 2′ position enables more effective π conjugation between these moieties, increasing delocalisation of the highest occupied molecular orbitals across the P-ring oxadiazole segment and stabilising the lowest unoccupied molecular orbitals. This stabilisation slightly reduces the HOMO–LUMO energy gap, leading to lower energy electronic transitions and the observed red shift. In contrast, when the nitrogen atom occupies the 3′ position in MTPO-3, conjugative overlap between the P-ring and the oxadiazole core is weakened. This reduced electronic communication results in a more localised frontier orbital distribution and a marginally higher excitation energy, consistent with the shorter wavelength absorption and reduced overall intensity observed for MTPO-3. The weaker participation of the P-ring in the HOMO → LUMO transition is also reflected in its slightly lower oscillator strength relative to MTPO-2. As a result, these spectral features indicate that positional isomer dependent modification of the P-ring primarily tunes the intensity and band shape of the low energy electronic transitions, with the accompanying λ_max_ shift representing a smaller, secondary effect, without altering the fundamental conjugated scaffold.

**Fig. 6:**
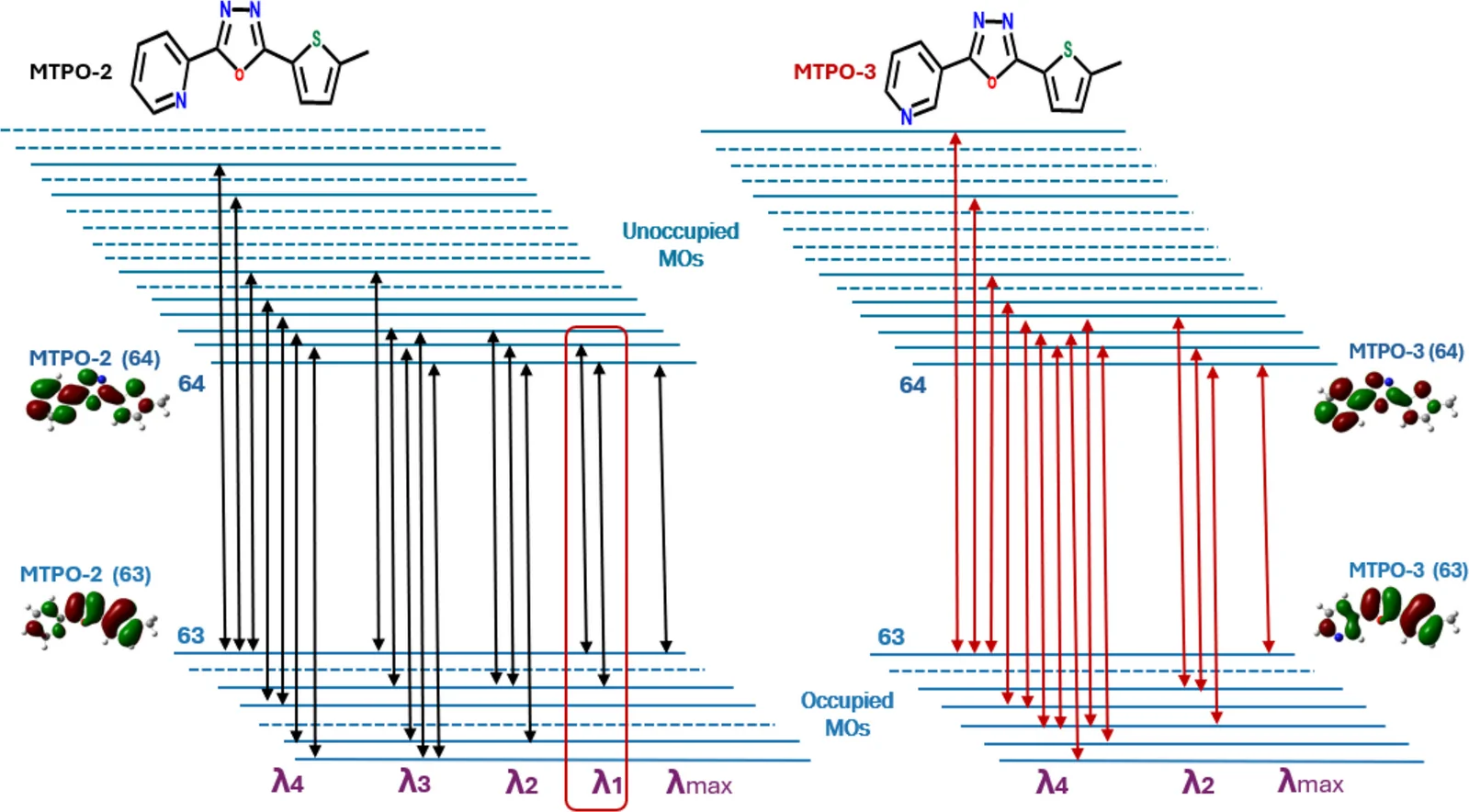
Comparison of the calculated UV–Vis spectral transitions of MTPO-2 (left) and MTPO-3 (right) in CHCl_3_ solvent using B3LYP/6–311 ++ G (d,p)

To understand the origin of the additional absorption features observed in MTPO-2, the singlet–singlet electronic transitions (up to 60 states) contributing to the UV–Vis spectra were analysed in terms of the frontier molecular orbitals, which are given in Fig. 6. For both MTPO-2 and MTPO-3, the lowest energy absorption (λ_max_) is dominated by a highest occupied molecular orbital (HOMO) and lowest unoccupied molecular orbital (LUMO), i.e. the HOMO → LUMO transition with partial charge transfer (CT) character. In both isomers, the HOMO is primarily localised on the O-ring and T-ring (Table 2), whereas the LUMO exhibits significant contributions from the P-ring together with the oxadiazole unit. As a result, differences in the electronic structure of the P-ring directly affect the LUMO energy and spatial distribution and, therefore, exert a strong influence on the excitation energy associated with λ_max_.

**Table 2:**
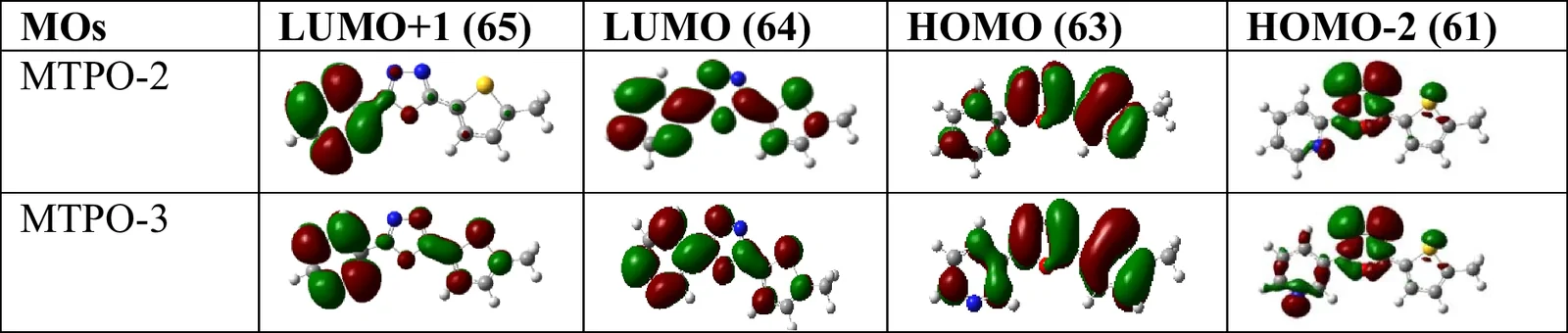
Frontier orbitals of MTPO-2 and MTPO-3 responsible for λ_max_ (and λ_1_) transitions

In MTPO-2, relocation of the nitrogen atom to the 2′ position enhances electronic coupling between the P-ring and the oxadiazole core, leading to greater delocalisation of the LUMO across the P-ring–O-ring segment (see the HOMO and LUMO in Table 2). This stabilisation of the LUMO, while the HOMO remains largely unchanged, reduces the HOMO–LUMO energy gap contributing to the slight bathochromic shift observed for MTPO-2, influencing the electronic transition energies and oscillator strengths. In contrast, nitrogen placement at the 3′ position in MTPO-3 weakens P-ring participation in the LUMO, resulting in a more localised acceptor orbital and a higher energy HOMO → LUMO transition.

Beyond the lowest energy transition, additional absorption bands (λ_1_ and λ_3_) are observed exclusively in MTPO-2, representing a key spectroscopic distinction between the two positional isomers. In particular, the absorption band at approximately 281 nm (λ_1_) for HOMOLUMO + 1 (63–65) and HOMO-2LUMO (61–65), arises from excited states with pronounced n → π* character, involving heteroatom lone pair orbitals on the O-ring and T-ring as donors and unoccupied orbitals with substantial P-ring character as acceptors. The enhanced delocalisation and favourable orbital alignment in MTPO-2 enable these n → π* transitions to carry appreciable oscillator strength. The related states of λ_max_ and λ_1_ are provided in Table S2. The absorption band at 192 nm (λ_3_) contain multiple frontier orbitals of MTPO-2 (refer to Table S2 in Supplementary Materials). In MTPO-3, nitrogen repositioning disrupts the geometric overlap between the lone-pair donor orbitals and the P-ring dominated acceptor orbitals, suppressing the corresponding transitions and rendering λ_1_ and λ_3_ effectively absent from the spectrum.

At higher energies, both MTPO-2 and MTPO-3 exhibit absorption bands in the deep UV region (e.g. λ_2_ around 224 nm), which arise from multiple closely spaced singlet–singlet excitations with mixed π → π* and n → π* character distributed across the molecular framework. These higher energy transitions involve more localised orbital contributions and are therefore less sensitive to P-ring nitrogen position, leading to comparable excitation energies in both positional isomers. Additional bands predicted near 183 nm originate from higher lying excited states composed of multiple electronic transitions and fall outside the experimentally accessible range.

In summary, the singlet–singlet transition analysis demonstrates that P-ring nitrogen repositioning selectively enables additional n → π* and CT-type excited states in MTPO-2, giving rise to λ_1_ and λ_3_ bands that are absent in MTPO-3. While the HOMO remains dominated by the O-ring and T-ring in both isomers, differences in P-ring participation in the LUMO manifold play a decisive role in shaping the UV–Vis spectral fingerprints of the two positional isomers. This frontier orbital imbalance explains the observed variations in spectral features and provides a direct link to the ionisation potential analysis that follows, where the occupied electronic states relevant to iron surface interactions are examined.

### Ionisation spectra

While UV–Vis spectroscopy elucidates excited-state behaviour, ionisation potentials (IP) provide direct insight into the ground state occupied orbitals responsible for electron donation. In this section, the IPs of MTPO-2 and MTPO-3 are first evaluated quantitatively using OVGF and Hartree–Fock (HF) calculations, before examining their corresponding IP spectra. Although the complete OVGF spectrum captures the full manifold of occupied orbitals, the first ionisation potential (IP), which is closely related to the HOMO energy, is commonly used as an electronic descriptor of a molecule’s intrinsic electron donating ability and is therefore an important consideration in corrosion inhibitor design. It reflects frontier orbital reactivity that may influence subsequent inhibitor–surface interactions. The IPs of a molecule can be estimated using the Koopman Theorem, which estimates ionisation energies of a molecule as the negative of the orbital energies obtained from the HF calculations. However, for Kohn–Sham DFT calculations, only the first IP is the negative of the HOMO energy, which is Janak Theory (Janak 1978), as Koopman Theorem does not work for DFT orbital energies. However, DFT orbital energies are employed to estimate IPs for molecules through meta-Koopman Theorem for convenience (Abeng et al. 2025; Benchinoune et al. 2026).

**Fig. 7:**
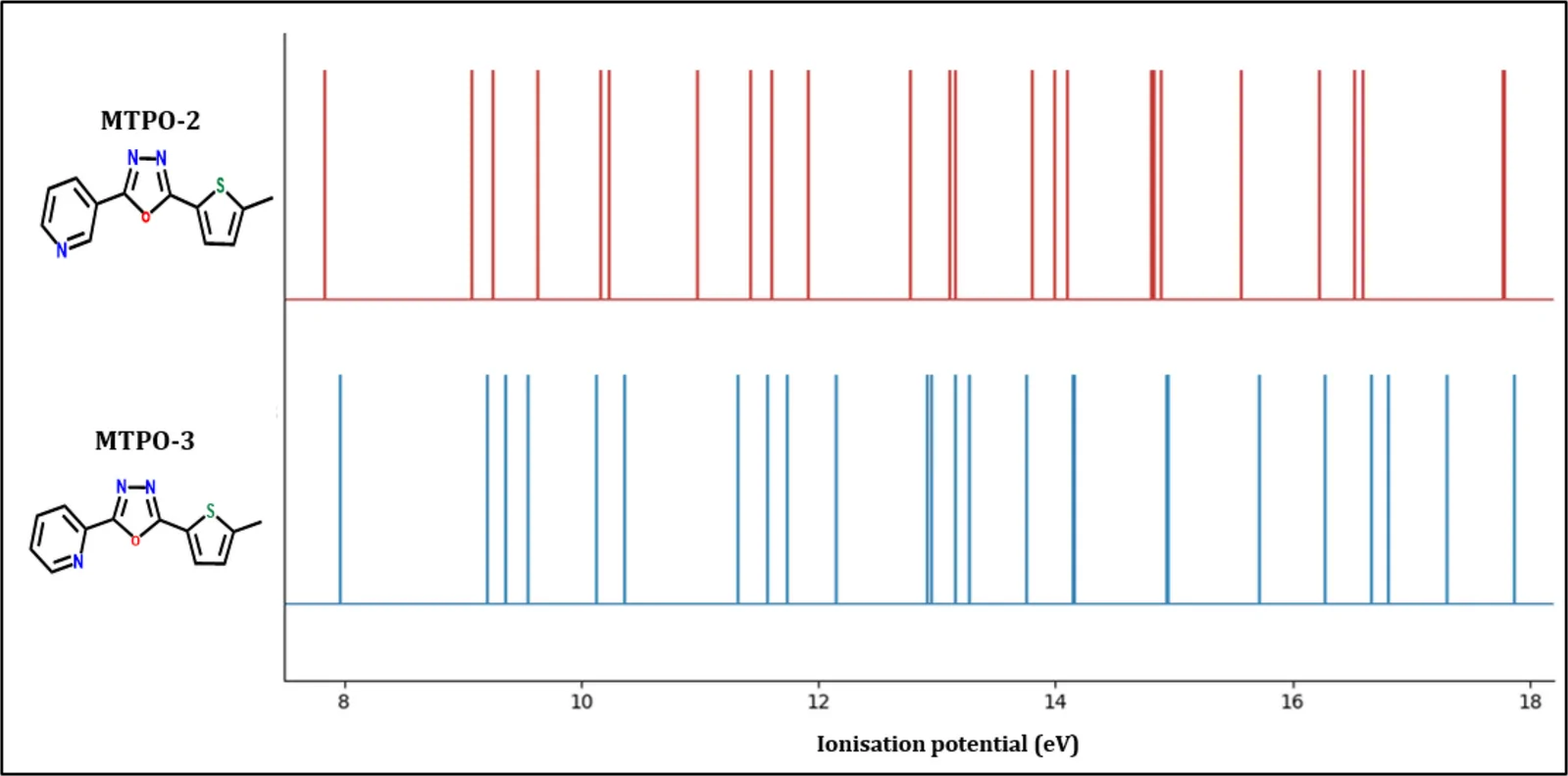
Comparison of ionisation spectra of MTPO-2 and MTPO-3 in the outer valence space using OVGF/6–311 ++ G level of theory. The detailed IPs are listed in Table S3 of the Supplementary Materials

To obtain more reliable IP estimates, the outer valence green’s function (OVGF) method (von Niessen et al. 1984) was employed, which accurately predicts IPs of isolated molecules (Wang and Ganesan 2014; Casier et al. 2021). Figure 7 presents the calculated outer valence IPs of MTPO-2 and MTPO-3 using the OVGF/6–311 ++ G level of theory. The calculated energies used to produce this figure are provided in Table S3. The OVGF ionisation potential spectra of MTPO-2 and MTPO-3 span the outer valence region from approximately 7.8 to 18 eV and display a high degree of overall similarity. Both spectra contain a comparable number of ionisation bands with similar clustering patterns, reflecting the shared thiophene–oxadiazole framework and identical molecular composition of the two positional isomers. This similarity is particularly evident in the positions of the first ionisation bands, where the lowest IPs of MTPO-2 and MTPO-3 occur at closely related energies of 7.83 eV for MTPO-2 and 7.97 eV for MTPO-3, indicating that the HOMOs (Table S3) in both isomers are of similar energetic character and chemical origin.

The two stick spectra span ~ 7.8–17.9 eV and show similarities in band density and clustering, consistent with their shared thiophene–oxadiazole scaffold and closely related π framework. Despite this, the spectra exhibit differences in individual IP positions across the entire valence region. The differences in IP band positions can be directly attributed to nitrogen repositioning in the P-ring. Moving the nitrogen atom from the 3′ position in MTPO-3 to the 2′ position in MTPO-2 modifies the electron-withdrawing character and conjugative interaction of the P-ring with the oxadiazole unit. Because the molecule forms an extended conjugated aromatic system, this local structural change propagates through the π-framework and redistributes electron density across the entire molecule. As a result, the energies of multiple occupied orbitals are perturbed, leading to the observed structure dependent shifts and band reordering in the IP spectrum.

The IP spectra show that MTPO-2 and MTPO-3 are closely related but not electronically equivalent. Their similar first IPs and low RMSD (0.175 eV) confirm a shared electronic framework, while the consistent differences in band positions demonstrate that P-ring positional isomerism subtly reshapes the occupied electronic structure through conjugation. This occupied state perspective complements the UV–Vis analysis, which highlights differences in unoccupied orbitals and excited states, and together they provide a coherent picture of how nitrogen repositioning influences the electronic properties of MTPO isomers.

## Conclusion

This work demonstrates that subtle structural variations within heteroaromatic corrosion inhibitors can be exploited as an effective electronic structure tuning strategy. Using MTPO-3 as a reference system, systematic DFT calculations identify the pyridine-2-yl positional isomer (MTPO-2) as the lowest energy isomer (by 0.57 kJ · mol^−1^), indicating that nitrogen repositioning within the pyridine ring (P-ring) is not only chemically viable but energetically favourable. This finding highlights the importance of considering positional isomeric forms during inhibitor screening, rather than treating reported structures as electronically unique.

By integrating ground and excited state spectroscopic descriptors within a single computational framework, the study clarifies how P-ring modification propagates through the conjugated aromatic system to reshape both occupied and unoccupied electronic states. NMR analyses show that positional isomerism primarily perturbs the electronic environment of the pyridine ring, while preserving the thiophene–oxadiazole backbone. UV–Vis and TD-DFT results further reveal that this local structural change selectively stabilises LUMO-dominated states, leading to small red-shifted absorption, increased transition probability, and the emergence of additional n → π* features unique to MTPO-2. These effects arise because the HOMO remains largely invariant across isomers, whereas the LUMO manifold is highly sensitive to pyridine-ring electronic structure, imparting partial charge transfer character to the lowest energy excitations.

Ionisation potential (IP) spectra provide a complementary, occupied state perspective that is rarely addressed in corrosion inhibitor studies. The strong correlation between the IP spectra of MTPO-2 and MTPO-3 confirms a shared electronic framework, while systematic, non-uniform shifts across the valence region show that nitrogen repositioning perturbs multiple occupied orbitals through conjugated aromatic coupling. This behaviour demonstrates that positional isomerism reshapes the entire valence electronic landscape rather than producing a trivial frontier orbital shift, reinforcing the value of IP analysis as a discriminator between closely related inhibitor candidates.

From a design standpoint, the results establish pyridine-ring positional isomerism as a practical molecular lever for tuning electronic descriptors relevant to corrosion inhibitor design. The combined use of NMR, UV–Vis, and IP analyses provides a physically transparent route to connect local structural changes with global electronic response. While adsorption and surface-specific effects are not addressed here, the electronic structure insights developed in this work provide a rational basis for selecting and prioritising MTPO-type inhibitors for subsequent surface interaction and performance studies.

## Supplementary information

Below is the link to the electronic supplementary material.

ESM 1 (DOCX 131 KB)

## Data Availability

Data will be available on request.
