# Supplementary material for: Computational investigation of a new pyridin-2-yl isomer of thiophene–pyridine 1,3,4-oxadiazole (MTPO) for steel corrosion inhibitor design
Source: Environ Sci Pollut Res Int. 2026 Oct 30;33(40):18077–90. doi: 10.1007/s11356-026-38362-y (PMC13769694; doi:10.1007/s11356-026-38362-y)
Supplement: Supplementary file 1 [file 11356_2026_38362_MOESM1_ESM.docx]

**Supplementary Information**

**Computational investigation of a new pyridin‑2‑yl isomer of thiophene–pyridine 1,3,4‑oxadiazole (MTPO) for steel corrosion inhibitor design**

Heshani Balasooriya^1^, Chunqing Li^2^, Feng Wang^1^*

^1^School of Science, Computing and Emerging Technologies, Swinburne University of Technology, Melbourne, Victoria 3122, Australia

^2^School of Engineering, RMIT University, Melbourne, Vic 3000, Australia

Correspondence: [fwang@swin.edu.au](mailto:fwang@swin.edu.au)

**Table S1.** Summary of corrosion inhibition performance of various oxadiazole-based compounds for mild steel under different experimental conditions

| **No** | **Name of the inhibitor** | **Molecular Structure** | **Inhibitor code** | **Exp conditions*** | **Inhibition efficiency (%)** | **Ref** |
| --- | --- | --- | --- | --- | --- | --- |
| 1 | 5-(6-chloropyridin-3-yl)-*N*-(2,6-difluorophenyl)-1,3,4-oxadiazol-2-amine |  | OXDA1 | 10⁻³ M  1M HCl | 88.66% | (Üstün, et al. 2025) |
| 2 | 5-(6-chloropyridin-3-yl)-*N*-(2,6-dimethylphenyl)-1,3,4-oxadiazol-2-amine |  | OXDA2 | 10⁻³ M  1M HCl | 95.02% | (Üstün, et al. 2025) |
| 3 | N-((5‑chloro-3-methyl-1-phenyl-1H-pyrazol-4-yl)methylene)−5-(4-methoxyphenyl)−1,3,4-oxadiazol-2-amine |  | CMPPOA | 300 ppm  1M HCl  298 K | 90.12% | (Khanna, et al. 2024) |
| 4 | 2-(4-methoxyphenyl)-5-(pyridin-3-yl)-1,3,4-oxadiazole |  | 4-PMOX | 500 ppm  1N HCl  303 K | 98.57% | (Sharma, et al. 2023) |
| 5 | 2,5-disubstituted oxadiazole derivatives, namely, 2-benzyl-5-(4nitrophenyl)-1,3,4-oxadiazole |  | 2B54NPO | 300 ppm  1M HCl  298 K | 94.08% | (Kumar, et al. 2022) |
| 6 | 2-(4-methoxyphenyl)-5-(phenoxymethyl)-1,3,4-oxadiazole |  | 24MO5POO | 300 ppm  1M HCl  298 K | 92.37% | (Kumar, et al. 2022) |
| 7 | 2-N-phenylamino-5-(3-phenyl-3-oxo-1-  propyl)-1,3,4-oxadiazole |  | NPOPO | 500 ppm  1M HCl  303 K | 95.1 % | (Alamiery 2021) |
| 8 | 2-(5-methylthiophen-2-yl)-5-(pyridin3-yl)-1,3,4-oxadiazole |  | MTPO | 500 ppm  1N HCl  298 K | 99.05% | (Sharma, et al. 2025) |

*Experimental conditions include inhibitor concentration/medium/temperature

**Table S2.** Transitions of UV-vis spectra of AS2 and AS3

| **_UV-Vis Band_** | **_AS2_** | | **_AS3_** | | |
| --- | --- | --- | --- | --- | --- |
|  | **_Position (nm)_** | **_Transition_** | **_Position (nm)_** | **_Transition_** | **_Expt (nm)#_** |
| _λmax_ | _345.00 (ES 1)_ | _63🡪64 (0.8291)_ | _341.71(ES 1)_ | _63🡪64 (0.8161)_ | _315_ |
| _λ1_ | _281.62 (ES 2)_ | _61🡪64 (0.1072)_  _63🡪65_ |  |  |  |
| _λ2_ | _224.29 (ES 14)_ | _58🡪64 (0.1212)_  _61🡪65_  _61🡪66_ | _224.01(ES 14)_ | _59🡪64 (0.1727)_  _61🡪65_  _61🡪66_ | _240_ |
| _λ3_ | _192.20 (ES 27)_ | _57🡪64 (0.2762)_  _57🡪66_  _58🡪65_  _61🡪66_  _63🡪70_ |  |  |  |
| _λ4_ | _183.50 (ES 35)_ | _57🡪65 (0.1329)_  _58🡪66_  _60🡪67_  _60🡪68_  _63🡪70_  _63🡪75_  _63🡪77_ | _183.41 (ES 36)_ | _57🡪64 (0.3291)_  _57🡪65_  _57🡪66_  _59🡪65_  _59🡪66_  _60🡪67_  _60🡪68_  _63🡪70_  _63🡪75_  _63🡪79_ |  |

********Calculated using DFT/B3LYP/6-311G++(d,p) in CHCl_3_ solvent*

**Table S3.** OVGF Orbital Ionization Potentials and Pole Strengths for AS2 and AS3

| **Orbital** | **AS1** | | **AS2** | |
| --- | --- | --- | --- | --- |
|  | **Ionization potential (eV)** | **Pole strength (PS)** | **Ionization potential (eV)** | **Pole strength (PS)** |
| 17 | 17.788 | 0.876 | 17.864 | 1.062 |
| 18 | 17.780 | 0.007 | 17.299 | 0.859 |
| 19 | 16.598 | 0.804 | 16.810 | 0.802 |
| 20 | 16.525 | 0.862 | 16.666 | 0.861 |
| 21 | 16.224 | 0.868 | 16.270 | 0.867 |
| 22 | 15.564 | 0.873 | 15.719 | 0.875 |
| 23 | 14.887 | 0.881 | 14.940 | 0.881 |
| 24 | 14.807 | 0.891 | 14.949 | 0.890 |
| 25 | 14.830 | 0.869 | 14.938 | 0.868 |
| 26 | 14.098 | 0.880 | 14.143 | 0.880 |
| 27 | 13.996 | 0.892 | 14.161 | 0.891 |
| 28 | 13.803 | 0.819 | 13.762 | 0.821 |
| 29 | 13.150 | 0.892 | 13.277 | 0.892 |
| 30 | 13.112 | 0.894 | 13.155 | 0.894 |
| 31 | 12.777 | 0.851 | 12.955 | 0.851 |
| 32 | 12.783 | 0.903 | 12.916 | 0.902 |
| 33 | 11.427 | 0.867 | 11.577 | 0.867 |
| 34 | 11.912 | 0.861 | 12.148 | 0.860 |
| 35 | 11.609 | 0.897 | 11.735 | 0.896 |
| 36 | 10.162 | 0.870 | 10.371 | 0.869 |
| 37 | 10.986 | 0.873 | 11.323 | 0.871 |
| 38 | 9.639 | 0.883 | 9.550 | 0.885 |
| 39 | 10.243 | 0.886 | 10.130 | 0.886 |
| 40 | 9.263 | 0.894 | 9.211 | 0.902 |
| 41 | 9.082 | 0.901 | 9.358 | 0.893 |
| 42 | 7.834 | 0.888 | 7.970 | 0.887 |

**Using 6-311G++.*

**References**

Alamiery A (2021) Corrosion inhibition effect of 2-N-phenylamino-5-(3-phenyl-3-oxo-1-propyl)-1,3,4-oxadiazole on mild steel in 1 M hydrochloric acid medium: Insight from gravimetric and DFT investigations. Mater Sci Energy Technol 4:398–406. <https://doi.org/10.1016/j.mset.2021.09.002>

Khanna R, Dudi M, Mangla B, Kalia V, Sihmar A, Tanwar H, Dahiya H (2024) Assessment of pyrazole Schiff base's corrosion inhibition effectiveness incorporating oxadiazole moiety on mild steel in 1 M HCl: A holistic theoretical and experimental analysis. J Mol Struct 1317:139066. <https://doi.org/10.1016/j.molstruc.2024.139066>

Kumar S, Kalia V, Goyal M, Jhaa G, Kumar S, Vashisht H, Dahiya H, Quraishi MA, Verma C (2022) Newly synthesized oxadiazole derivatives as corrosion inhibitors for mild steel in acidic medium: Experimental and theoretical approaches. J Mol Liq 357:119077. <https://doi.org/10.1016/j.molliq.2022.119077>

Sharma D, Thakur A, Sharma MK, Sharma R, Kumar S, Sihmar A, Dahiya H, Jhaa G, Kumar A, Sharma AK, Om H (2023) Effective corrosion inhibition of mild steel using novel 1,3,4-oxadiazole-pyridine hybrids: Synthesis, electrochemical, morphological, and computational insights. Environ Res 234:116555. <https://doi.org/10.1016/j.envres.2023.116555>

Sharma D, Thakur A, Sharma MK, Bhardwaj A, Sihmar A, Dahiya H, Sharma AK, Kumar A, Berisha A, Om H (2025) Experimental and computational studies on the corrosion inhibition potential of a novel synthesized thiophene and pyridine-based 1,3,4-oxadiazole hybrid against mild steel corrosion in 1 N HCl. Environ Sci Pollut Res 32:17005–17031. <https://doi.org/10.1007/s11356-024-32678-3>

Üstün N, Dalouh M, Azghay I, Zannagui C, Chiban M, Lahhit A, Aziam R, Elyoussfi A, Ahari Mh, Salhi A, Tok F (2025) Corrosion inhibition and substituent impact of newly synthesized 1,3,4-oxadiazole derivatives on mild steel in acidic media: Electrochemical and quantum chemistry insights. J Mol Struct 1341:142685. <https://doi.org/10.1016/j.molstruc.2025.142685>
